# Supplementary figures and images for: Distribution and Phylogenetic Diversity of Synechococcus-like Cyanobacteria in the Late Autumn Picophytoplankton of the Kara Sea: The Role of Atlantic and Riverine Water Masses
Source: Plants (Basel). 2025 Aug 22;14(17):2614. doi: 10.3390/plants14172614 (PMC12429990; doi:10.3390/plants14172614)

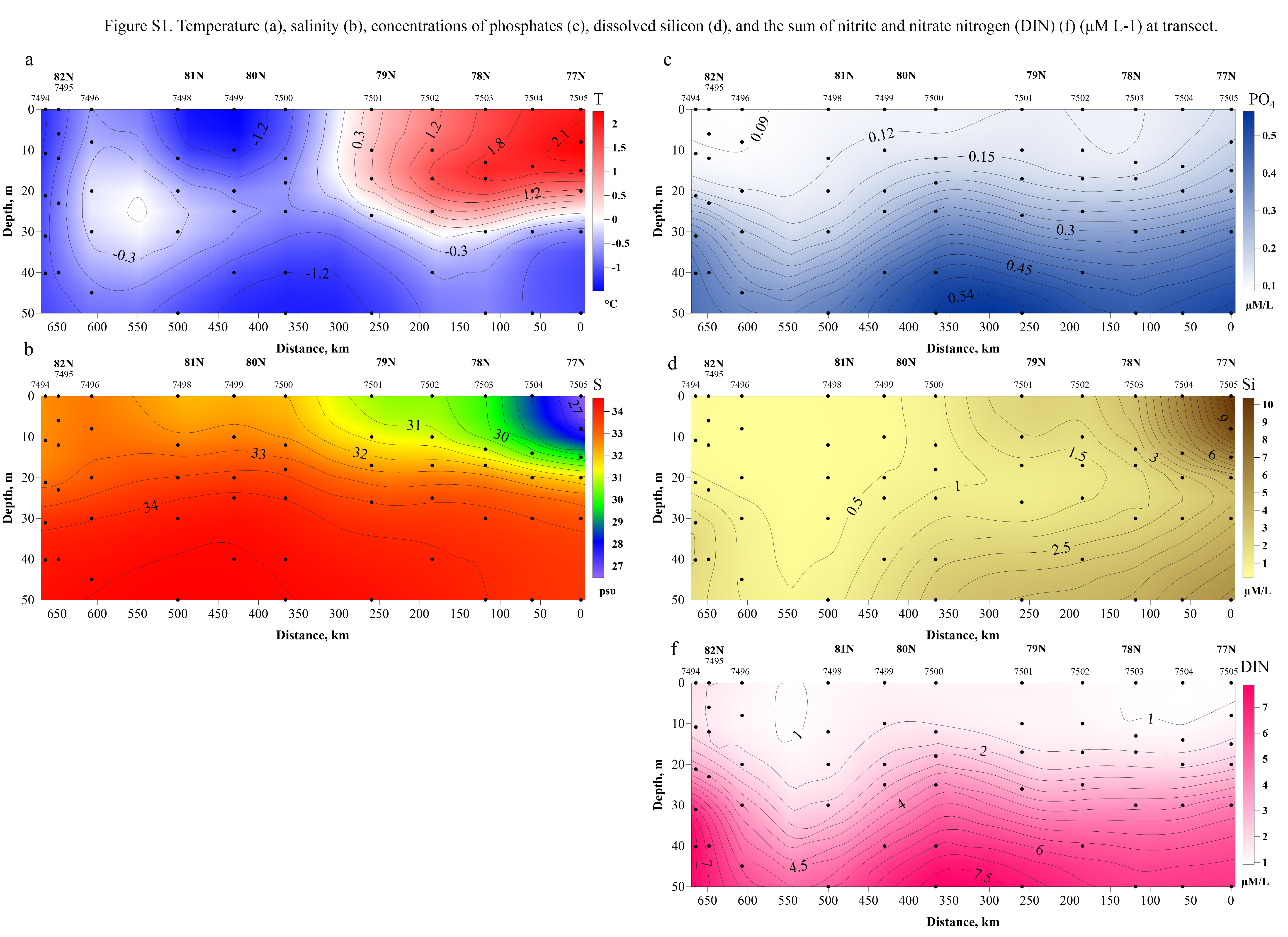

Supplement: Supplementary file 1 [file plants-14-02614-s001.zip › Figure S1-1.jpg]
